# Quality of life before and after catheter ablation (pulmonary vein isolation) for atrial fibrillation: Results from the Netherlands Heart Registration

**DOI:** 10.1007/s12471-025-02014-6

**Published:** 2026-01-19

**Authors:** Tom Oirbans, Jonas S. S. G. de Jong, Gijs J. van Steenbergen, Ahmet Adiyaman, Bas A. Schoonderwoerd, Hilda G. Rijnhart-de Jong, Pepijn H. van der Voort, Justin G. L. M. Luermans, Sjoerd W. Westra, Wichert J. Kuijt, Michelle D. van der Stoel, Johannes C. Kelder, Lucas V. A. Boersma, Jippe C. Balt

**Affiliations:** 1 Department of Value-Based Healthcare, St. Antonius Hospital, Nieuwegein, The Netherlands; 2 Department of Cardiology, OLVG, Amsterdam, The Netherlands; 3 Department of Cardiology, Catharina Hospital, Eindhoven, The Netherlands; 4 Department of Cardiology, Isala Hospital, Zwolle, The Netherlands; 5 Department of Cardiology, Medical Center Leeuwarden, Leeuwarden, The Netherlands; 6 Department of Cardiology, Cardiovascular Research Institute Maastricht (CARIM), Maastricht University Medical Center (MUMC+), Maastricht, The Netherlands; 7 Department of Cardiology, Radboud Medical Center, Nijmegen, The Netherlands; 8 Department of Cardiology, Amphia Hospital, Breda, The Netherlands; 9 Netherlands Heart Registration, Utrecht, The Netherlands; 10 Department of Cardiology, St. Antonius Hospital, Nieuwegein, The Netherlands

**Keywords:** Electrophysiology, Atrial fibrillation, Radiofrequency ablation, Quality registry, Value-based healthcare

## Abstract

**Background:**

Reducing AF-related symptoms and improving health-related quality of life (HRQoL) are important drivers in the decision for pulmonary vein isolation (PVI) in treating symptomatic atrial fibrillation (AF). We assessed the association between various patient characteristics, intervention, and outcome variables, and HRQoL both prior to and one year after PVI, with specific attention to groups that did not improve or were still impaired in HRQoL post PVI.

**Methods:**

Observational, retrospective multicenter cohort study within 8 hospitals participating in the Netherlands Heart Registration (NHR). Patients who underwent PVI between 2016 and 2019 and completed the Atrial Fibrillation Effect on Quality-of-Life (AFEQT) questionnaire both prior to and one year after were included. Accepted cut-off values for impaired HRQoL and clinically important difference (CID) were used.

**Results:**

Mean AFEQT score (*n* = 2,534) was 55.6 ± 19.7 prior to intervention and 79.8 ± 20.2 after. Post-PVI, 39.5% of the population was still impaired in HRQoL (< 80 points), and 19.2% failed to achieve CID (delta ≥ 5 points). Lower baseline AFEQT-score (odds ratio [OR], 0.96 [per 1‑point increase]; 95% CI, 0.96–0.97; *p* < 0.001) and female sex (odds ratio [OR], 1.42; 95% CI, 1.16–1.75; *p* < 0.001) were the most prominent related factors with impaired HRQoL post-PVI. Higher baseline AFEQT-score (odds ratio [OR], 1.04 [per 1‑point increase]; 95% CI, 1.04–1.05; *p* < 0.001) was strongly associated with failure to achieve CID.

**Conclusion:**

Despite a major increase in HRQoL across the population, over one-third of patients were still impaired in HRQoL post-PVI. Multiple factors were identified that may guide counselling of AF patients about treatment choice.

**Supplementary Information:**

The online version of this article (10.1007/s12471-025-02014-6) contains supplementary material, which is available to authorized users.

## What’s new?

Patients with low HRQoL prior to PVI have a relative high likelihood of achieving a clinically important difference, the chances of achieving a preserved HRQoL however is considerably smaller.

## Introduction

In the Netherlands, over 360,000 patients are diagnosed with atrial fibrillation (AF) [1]. AF can cause a variety of symptoms such as palpitations, fatigue, shortness of breath, and malaise, which can significantly impact the daily functioning and Quality of life of the patient (QoL) [2, 3]. Moreover, AF is associated through multiple, often complex, mechanisms with substantial morbidity [4, 5] and related healthcare consumption [6]. The treatment of AF is aimed at stroke prevention, reduction of AF-related symptoms, and improvement of QoL [7]. The severity of AF symptoms and the accompanying burden for patients should drive the decision for the treatment strategy [8]. Especially for symptomatic patients, this often translates into the so-called ‘rhythm control strategy’; restore and maintain sinus rhythm along with adequate rate control, anticoagulation therapy, and lifestyle counselling. The current European Society of Cardiology (ESC) guideline states that the primary indication for rhythm control is to reduce AF-related symptoms and improve QoL. When a first-line strategy of anti-arrhythmic drugs is not sufficient in restoring and maintaining sinus rhythm, there is a class 1 recommendation for pulmonary vein isolation (PVI) [9]. During PVI, the pulmonary veins are isolated around their antrum by generating lesions by either radiofrequency or freezing. The success of the procedure is traditionally expressed in the percentage of patients with freedom from AF after PVI. This rate from freedom of AF, however, largely depends on the intensity of monitoring and duration of follow-up, and varies among studies [10]. Classifying procedure success solely by the complete absence of AF does not consider that reduction of duration and intensity of AF episodes can have positive effects on the health status of the patient. An alternative way of assessing treatment success, in line with patient-centered care [11] and current guidelines [9], is from the patient’s perspective by using patient-reported outcomes (PROs) like Health Related Quality of Life (HRQoL). The International Consortium for Health Outcomes Measurement (ICHOM) AF working group has proposed tools for assessing HRQoL in their standard set of outcomes measures [12]. To assess exercise tolerance and symptom severity, ICHOM advises using the Atrial Fibrillation Effect on Quality-of-Life (AFEQT) Questionnaire, a commonly used and validated instrument to determine AF-related quality of life [13]. At the population level, the benefits of PVI on quality of life scores expressed by the AFEQT questionnaire have been demonstrated in several studies [14, 15]. Although the average population effect seems impressive, up to a third of patients do not benefit from PVI in terms of HRQoL improvement [16]. Identifying subgroups or certain characteristics associated with non-improvement in HRQoL after PVI can be helpful in patient selection and shared decision making, as the indication for PVI for AF is, in part, driven by HRQoL improvement [9]. In this observational, retrospective multicenter cohort study, we assessed the relationship between different patient characteristics, intervention, and outcome variables and AF-related quality of life in patients who underwent PVI for AF. This includes the development of a predictive model and multiple subgroup analyses with specific attention to those groups that did not improve or were still impaired in HRQoL after PVI.

## Methods

We used data from The Netherlands Heart Registration (NHR), a non-profit organisation facilitating high-quality registration of percutaneous and surgical cardiac procedures in the Netherlands [17].

### Study population

Patients who underwent a PVI between January 2016 and December 2019 in one of the 8 participating centers in the Netherlands and completed the AFEQT questionnaire both before and one year after PVI were included.

### Baseline characteristics, quality of life assessment, and clinical outcomes

Baseline characteristics, interventional variables, and outcome variables were included for analysis. Completed AFEQT questionnaires, both prior to the PVI and one year after the PVI, were transformed to a 100-point AFEQT overall summary score (AFEQT-OS) scale, with a score of 0 corresponding to total disability and a score of 100 corresponding to no disability. Accepted cut-off values in AFEQT-OS scores, both for a score at a given moment as well as the significance of a change in score over time, were applied. A score of ≥ 80 points for preserved HRQoL and < 80 points for impaired HRQoL [16, 18, 19] and a 5-point change in the 1‑year AFEQT-OS score for a clinically important difference (CID) [20]. As the NHR database did not collect data on the maintenance of sinus rhythm post-PVI, recurrence of atrial fibrillation (AF) could not be included as an outcome variable.

### Statistical methods

Descriptive variables were reported as frequencies and percentages for categorical variables and as median with interquartile range (IQR) or mean with standard deviation (SD) for continuous variables. To define factors independently associated with the AFEQT-OS score at baseline, a multiple linear regression model was constructed. Quartiles for AFEQT-OS score at baseline were compared for differences in case-mix using Pearson’s χ2 test (categorical variables) and for continuous variables, one-way analysis of variance (ANOVA). The difference between the one-year and the baseline overall AFEQT summary score (delta AFEQT-OS) was evaluated with the Student’s paired t tests (95% CI). Additionally, a multivariable generalized linear model with clinically relevant covariates (including the AFEQT-OS score at baseline) was used to predict the AFEQT-OS score at 1 year after PVI. We used accepted cutoff values for CID (a 5-point change in the 1‑year AFEQT-OS score) and impaired HRQoL (AFEQT-OS score < 80 points) as outcomes. First, we explored the rates of patients within subgroups and compared these using χ2 tests. Secondly, logistic regression models were constructed using backward selection with a stepwisecriterion of 0.05.

## Results

In total, 10,536 patients underwent a PVI in the period from January 2016 through December 2019 in the 8 participating centers. 2,534 patients (24%) completed both the baseline and the 1‑year follow-up AFEQT questionnaire and were used for further analysis (Fig S1). We found no major differences in baseline characteristics across the groups of patients with and without complete AFEQT data (Tab S1).

### Study population

Within the study cohort, 65.7% of the patients were male, and most had normal ejection fraction (87.1%), low CHA_2_DS_2_-VASc score (µ = 1), and paroxysmal AF (77.1%). About one-fifth of the population (22.3%) had a prior catheter ablation for atrial fibrillation in the past (Tab. 1).

**Table 1:** Baseline characteristics of evaluable cohort

Characteristics	Patients with AFEQT data (evaluable cohort) *N* = 2,534
*Patient Characteristics*	
Age, years median (Q1, Q3)	64 (57, 70)
< 65 years	*n* = 1,342 (53%)
≥ 65 to 74 years	*n* = 1,006 (39.7%)
≥ 75 years	*n* = 186 (7.3%)
Male Sex	*n* = 1,666 (65.7%)
BMI (kg/M^2^) Median (Q1, Q3)	27 (24, 29)
*Medical history*	
Left ventricular ejection fraction (LVEF) ≥ 50%	*n* = 2,039/2,341 (87.1%)
Pre-operative moderate/severe mitral valve regurgitation	*n* = 89/2,247 (4%)
CHA_2_DS_2_-VASc Median (Q1, Q3)	1 (1, 2)
– 0–1	*n* = 1,254/2,497 (50.3%)
– 2	*n* = 662/2,497 (26.5%)
– 3	*n* = 352/2,497 (13.9%)
– 4	*n* = 156/2,497 (6.2%)
– ≥ 5	*n* = 73/2,497 (2.9%)
Prior catheter ablation for AF	*n* = 558/2,497 (22.3%)
*Arrhythmia history*	
Type of AF at baseline	
Paroxysmal	*n* = 1,918/2,489 (77.1%)
Persistent/longstanding persistent	*n* = 571/2,489 (22.9%)
*Treatment strategy*	
Point-by-point	*n* = 1,076/2,487 (43.3%)
PVAC ± MASC/MAAC*	*n* = 499/2,487 (20.1%)
Cryo-ballon	*n* = 912/2,487 (36.7%)
Additional LA ablation	*n* = 306/2,340 (13.1%)

*MASC multi-array septal catheter, *MAAC* multi-array ablation catheter

### HRQoL prior to intervention

**Fig. 1:**
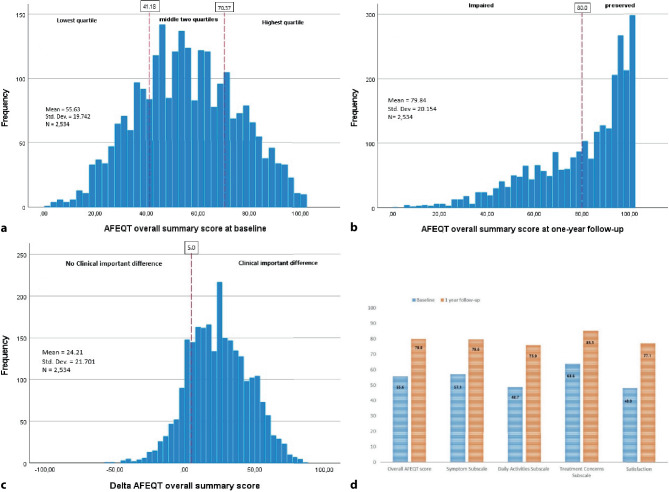
**a** Distribution of AFEQT score at baseline, including quartiles. **b** Distribution of AFEQT score at one year, including cut-off value for impairedness. **c** Distribution of Delta AFEQT score, including cut-off values for clinically important differences. **d** Change in mean AFEQT scores from baseline to one-year post procedure, including the domain-specific scores

Figure 1a displays the distribution of the AFEQT-OS score at baseline, including the quartiles as used. The mean score prior to intervention was 55.6 ± 19.7, with a large majority (87.5%) of patients reporting an impaired HRQoL (AFEQT-OS score < 80). In our multiple linear regression model we found female sex, higher Body Mass Index (BMI), reduced LVEF, persistent or longstanding persistent AF and higher CHA_2_DS_2_-VASc score all independently associated with lower baseline AFEQT-OS score (Tab. 2). In the distribution across quartiles of the AFEQT-OS baseline score, roughly the same covariables stood out, with the most notable being the breakdown to sex (Tab S2). Compared to women, a twice as large proportion of men were in the highest quartile of AFEQT-OS at baseline whereas women were overrepresented in the lowest quartile (Fig S2).

**Table 2:** Factors Independently Associated with AFEQT score at baseline

Factor	Results of a linear regression model			
	Estimated change from baseline	95% CI		*P* value
		Lower limit	Upper limit	
(Constant)	89,659	83,204	96,115	< 0.001
Female (vs male)	−7,724	−9,488	−5,960	< 0.001
BMI (per 1‑point increase)	−0.628	−0.819	−0.438	< 0.001
LVEF < 50 (vs ≥ 50)	−3,155	−5,525	−0.785	0.009
CHA_2_DS_2_-VASc (per 1 category increase)	−1,624	−2,401	−0.847	< 0.001
Persistent/longstanding atrial fibrillation (vs paroxysmal)	−0.250	−0.438	−0.063	0.009

### HRQoL one year after intervention

We found a mean one-year AFEQT-OS score of 79.8 ± 20.2, the domain-specific scores (symptoms, daily activities, and treatment concern) are shown in Fig. 1d. There was a significant increase in the difference between the one-year and the baseline AFEQT-OS score (delta AFEQT-OS) of 24.2 points, *p* < 0.001, 95% CI [23.4 to 25.1]. High HRQoL at baseline was the most prominent predictor for lower improvement, with a 1-point higher baseline AFEQT-OS score associated with a 0.6-point decrease, *p* 0.000, 95% CI [−0.661 to −0.583] in 1‑year AFEQT-OS score. The variables female sex, higher CHA_2_DS_2_-VASc score, higher BMI, and prior catheter ablation for AF were all associated with lower 1‑year AFEQT-OS score. On the contrary, the interventional characteristic of additional left atrial ablation was associated with an increase in 1‑year AFEQT-OS score (Tab. 3).

**Table 3:** Generalized linear model; Delta AFEQT is Dependent

Factor	Results of a Generalized linear model			
	Estimated change from baseline	95% CI		*P* value
		Lower limit	Upper limit	
(Constant)	68,965	63,123	74,808	0.000
Female (vs male)	−3,031	−4,735	−1,327	< 0.001
CHA_2_DS_2_-VASc 2 (vs 0–1)	−3,324	−5,154	−1,494	< 0.001
CHA_2_DS_2_-VASc 3 (vs 0–1)	−3,137	−5,470	−0.804	0.008
CHA_2_DS_2_-VASc 4 (vs 0–1)	−5,674	−8,897	−2,452	< 0.001
CHA_2_DS_2_-VASc ≥ 5 (vs 0–1)	−10,015	−14,427	−5,603	< 0.001
Additional LA ablation	3,913	1,598	6,238	< 0.001
Prior catheter ablation for AF	−3,826	−5,738	−1,915	< 0.001
BMI (per 1‑point increase)	−0.250	−0.433	−0.066	0.008
Baseline AFEQT summary score (per 1‑point increase)	−0.622	−0.661	−0.583	0.000

### Impaired quality of life after PVI and clinically important differences

Despite the major increase in the mean AFEQT-OS score after PVI (Tab S3), considerable differences in the population were found when broken down by cut-off values. One year after treatment, 39.5% of the population reported an impaired HRQoL, and 19.2% of the population showed no clinically important difference or CID (Fig. 1b, c). The distribution for several subgroups was drawn out in Tab. 4. More than half of the female participants were impaired in HRQoL one year after PVI (51.6%), compared to 33.2% of the men.

**Table 4:** Dichotomous AFEQT Outcomes at 1 Year Follow-up in Subgroups

	No. of patients (%)	Impaired HRQoL (AFEQT T1 < 80)		Clinically important difference (Delta AFEQT ≥ 5)	
		Impaired	Not impaired	No clinically important difference	Clinically important difference
*Total*					
Total population	2,534 (100)	1,001 (39.5)	1,533 (60.5)	486 (19.2)	2,048 (80.8)
*Subgroups*					
*Sex (N* *=* *2,534)*					
Male	1,666 (65.7)	*553 (33.2)*	*1,113 (66.8)*	327 (19.6)	1,339 (80.4)
Female	868 (34.3)	*448 (51.6)*	*420 (48.4)*	159 (18.3)	709 (81.7)
*Age (N* *=* *2,534)*					
< 65 years	1,342 (53)	*477 (35.5)*	*865 (64.5)*	*231 (17.2)*	*1,111 (82.8)*
≥ 65 to 74 years	1,006 (39.7)	*429 (42.6)*	*577 (57.4)*	*217 (21.6)*	*789 (78.4)*
≥ 75 years	186 (7.3)	*95 (51.1)*	*91 (48.9)*	*38 (20.4)*	*148 (79.6)*
*Ejection fraction (N* *=* *2,341)*					
≥ 50	2,039 (87.1)	*783 (38.4)*	*1,256 (61.6)*	382 (18.7)	1,657 (81.3)
< 50	302 (12.9)	*139 (46.0)*	*163 (54.0)*	63 (20.9)	239 (79.1)
*Pre-operative mitral valve insufficiently (N* *=* *2,247)*					
Non/mild	2,158 (96)	*834 (38.6)*	*1,324 (61.4)*	396 (18.4)	1,762 (81.6)
Moderate/severe	89 (4)	*45 (50.6)*	*44 (49.4)*	21 (23.6)	68 (76.4)
*CHA*_*2*_*DS*_*2*_*-VASc score (N* *=* *2,497)*					
0–1	1,254 (50.2)	*390 (31.1)*	*864 (68.9)*	217 (17.3)	1,037 (82.7)
2	662 (26.5)	*289 (43.7)*	*373 (56.3)*	135 (20.4)	527 (79.6)
3	352 (14.1)	*171 (48.6)*	*181 (51.4)*	66 (18.8)	286 (81.3)
4	156 (6.2)	*84 (53.8)*	*72 (46.2)*	37 (23.7)	119 (76.3)
≥ 5	73 (2.9)	*46 (63.0)*	*27 (37.0)*	23 (31.5)	50 (68.5)
*Prior catheter ablation for AF (N* *=* *2,497)*					
No	1,939 (77.7)	*732 (37.8)*	*1,207 (62.2)*	*346 (17.8)*	*1,593 (82.2)*
Yes	558 (22.3)	*247 (44.3)*	*311 (55.7)*	*131 (23.5)*	*427 (76.5)*
*Re-ablation during FU (N* *=* *2,531)*					
No	2,134 (84.3)	*756 (35.4)*	*1,378 (64.6)*	*355 (16.6)*	*1,779 (83.4)*
Yes	397 (15.7)	*243 (61.2)*	*154 (38.8)*	*130 (32.7)*	*267 (67.3)*
*Baseline quartile (N* *=* *2,534)*					
Lowest quartile ≤ 41.18	628 (24.8)	*378 (60.2)*	*250 (39.8)*	*58 (9.2)*	*570 (90.8)*
Two middle quartiles (41.67–69.79)	1,264 (49.9)	*518 (41.0)*	*746 (59.0)*	*217 (17.2)*	*1,047 (82.8)*
Highest quartile ≥ 70.37	642 (25.3)	*105 (16.4)*	* 537 (83.6)*	*211 (32.9)*	* 431 (67.1)*
*Type of AF at baseline (N* *=* *2,489)*					
Paroxysmal	1,918 (77.1)	743 (38.7)	1,175 (61.3)	359 (18.7)	1,559 (81.3)
Persistent/longstanding persistent	571 (22.9)	232 (40.6)	339 (59.4)	116 (20.3)	455 (79.7)
*Treatment strategy (N* *=* *2,487)*					
Point-by-point	1,076 (43.3)	*434 (40.3)*	*642 (59.7)*	210 (19.5)	866 (80.5)
Pvac ± Masc/Maac	499 (20.1)	*216 (43.3)*	*283 (56.7)*	82 (16.4)	417 (83.6)
Cryo-ballon	912 (36.7)	*327 (35.9)*	*585 (64.1)*	184 (20.2)	728 (79.8)
*Additional LA ablation (N* *=* *2,340)*					
No	2,034 (13.1)	820 (40.3)	1,214 (59.7)	*405 (19.9)*	*1,629 (80.1)*
Yes	306 (86.9)	111 (36.3)	195 (63.7)	*46 (15.0)*	*260 (85.0)*

**Fig. 2:**
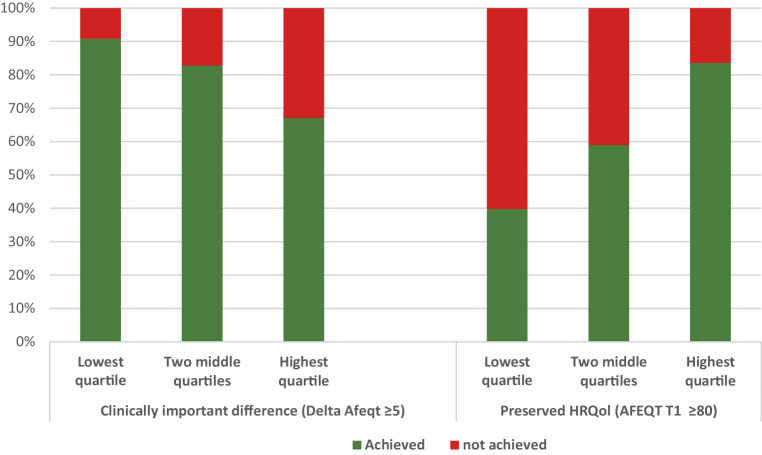
Percentages achieving clinically important difference (*CID*) or preserved HRQoL cutoff values one year after PVI per quartile for baseline AFEQT-OS score

Figure 2 shows the breakdown into quartiles of AFEQT-OS scores at baseline. We found patients in the lowest quartile were 3.5 times more likely to be impaired in HRQoL after PVI than those in the highest quartile (60.2% vs 16.4% of the patients, respectively). Contrastingly, the percentage of patients that did not reach the cutoff value for CID was much higher in patients in the highest quartile of AFEQT-OS score at baseline (32.9%) compared to the 9.2% in the lowest quartile.

Associations between sex and baseline AFEQT-OS scores on the dichotomized HRQoL outcomes remained after being adjusted for other variables in multivariable logistic regression (Tab S4).

## Discussion

In this Dutch multicenter registry with patients undergoing a PVI for AF, we studied the HRQoL with specific attention to those groups that did not improve or were still impaired one year after the intervention. The three main findings are: 1) A large majority of patients have an impaired HRQoL prior to PVI, with female sex and a higher CHA_2_DS_2_-VASc score being the most prominent associated factors. 2) Females and patients with low HRQoL at baseline were substantially more likely to have impaired HRQoL one year after the intervention. 3) A high baseline AFEQT-OS score is associated with a significantly smaller chance of reaching a clinically meaningful improvement in HRQoL one year after PVI.

### HRQoL prior to intervention

The HRQoL baseline score (55.6 ± 19.7) found in this study is consistent with other recent findings within this patient category [21, 22]. However, previous registry studies [16, 23, 24] based on the Keio interhospital Cardiovascular Studies-atrial (KiCS-AF registry) [19, 25] report AFEQT-OS baseline scores as much as 20 points higher. This remarkable difference might be partly due to differences in underlying health care systems and national legislation, as Ikemura et al. described [16]. A portion of the population within the KICS-AF registry was diagnosed with atrial fibrillation during a mandatory, annual health examination [26] and are therefore potentially less likely to be symptomatic compared to patients clinically diagnosed, resulting in a higher AFEQT-OS score at baseline. In line with multiple previous studies [22, 27, 28], we found female sex and a higher CHA_2_DS_2_-VASc score to be the most prominent baseline characteristics independently associated with lower AFEQT-OS score prior to intervention. Although there is no difference between men and women in overall prevalence of AF [29], women experience AF more often through atypical AF symptoms, which could result in a delay in diagnosis [22, 30] and referral for catheter ablation [31].

### HRQoL one year after intervention

We found a lower mean one-year AFEQT-OS score across our population (79.8 ± 20.2) than comparable conducted studies that report a mean of (well) above 80 points after one year [15, 16, 21, 23, 24, 32]. A percentage of 39.5% of our population did not reach the 80-point AFEQT-OS threshold at 1 year after treatment and were still impaired. The CABANA trial by Mark et al. [15] reported a 15% impairment rate 1 year after the procedure, similar to the KICS registry, where an impairment rate of 18.5% was found [16]. On the contrary, our patients experienced a considerably more positive clinically important difference or CID (a 5-point increase in the delta AFEQT-OS score) one year after PVI (80.8%), compared to 68.6% within the KICS-AF registry [16]. These differences might be largely explained by the lower baseline AFEQT-OS score of our population. As our study showed, patients in the lowest quartile AFEQT-OS score at baseline had a 3.5 times greater likelihood of being impaired at 1‑year, but on the other hand, were over three times more likely to reach the CID compared to those in the highest quartile. The rates of reaching CID are comparable to what was found within the KICS-AF registry, with the key difference that the reported baseline HRQoL was considerably higher in the latter. Consistent with other studies [16, 33], female sex was found to be an important factor in not reaching the 80-point threshold at 1 year. This difference is partly explained by the overrepresentation of women in the lowest quartile of HRQoL at baseline. Since the guidelines state that the primary indication for rhythm control is to reduce AF-related symptoms and improve QoL [9], the potential gain in HRQoL could be an important prerequisite in opting for a PVI. After all, when AF-related symptoms are relatively mild and the patient is not impaired in HRQoL, the chances of achieving a clinically important difference are slim. Even in patients with more severe AF-related symptoms and an impaired HRQoL, it is important to carefully consider the risks and gains. Although the likelihood of achieving a clinically important difference is high for these patients, the chances of achieving the cutoff value for preserved HRQoL are considerably smaller. The results of our study are broadly in line with findings from previous studies in this field. Disparities can be largely attributed to the relatively low baseline AFEQT-OS score of our population compared to some of the earlier work, although nation-specific agreements and practices also appear to play a role.

## Limitations

A limitation inherent to non-randomized observational studies: against the advantage of reporting real-world findings is the disadvantage of unmeasured variables with an impact on HRQoL that can cause a confounding effect. Further, due to heterogeneity in rhythm monitoring across centers (e.g., ECG, HolterHolter, or loop recorder), data on the maintenance of sinus rhythm post-PVI was insufficient. Consequently, atrial fibrillation recurrence was not included as an outcome variable. Lastly, the AFEQT questionnaire primarily captures symptom burden during the four weeks preceding the measurement, which may not fully reflect the episodic or fluctuating nature of AF-related complaints. This may have limited the ability to detect improvements in QoL, particularly among patients with higher baseline scores.

## Supplementary Information

Fig. S1. Flowchart of patient selection process

Fig. S2. AFEQT overall summary score at baseline across quartiles by Sex

Tab S1. Baseline characteristics for patients with/without AFEQT data

Tab S2 Differences between patients in the lowest- and highest quartile AFEQT at baseline

Tab S3: AFEQT en Delta AFEQT scores to quartile AFEQT at baseline

Tab S4: Factors independently associated with dichotomized HRQoL outcomes at one-year follow-up
