# Supplementary figures and images for: Quality of life before and after catheter ablation (pulmonary vein isolation) for atrial fibrillation: Results from the Netherlands Heart Registration
Source: Neth Heart J. 2026 Jan 19;34(2):72–9. doi: 10.1007/s12471-025-02014-6 (PMC12852550; doi:10.1007/s12471-025-02014-6)

Fig S 1


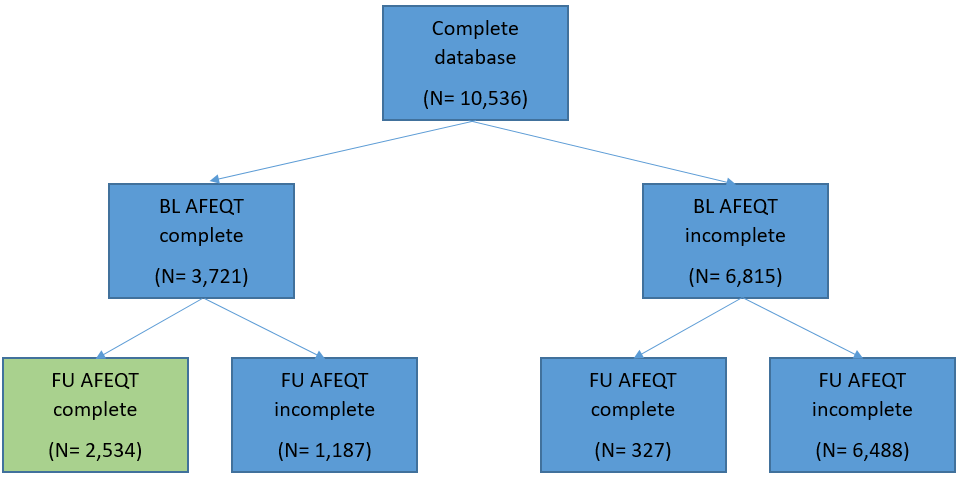


Flowchart of patient selection process

Supplement: Supplementary file 1 — Fig. S1. Flowchart of patient selection process [file 12471_2025_2014_MOESM1_ESM.docx]
