# Supplementary material for: Quality of life before and after catheter ablation (pulmonary vein isolation) for atrial fibrillation: Results from the Netherlands Heart Registration
Source: Neth Heart J. 2026 Jan 19;34(2):72–9. doi: 10.1007/s12471-025-02014-6 (PMC12852550; doi:10.1007/s12471-025-02014-6)
Supplement: Supplementary file 2 — Fig. S2. AFEQT overall summary score at baseline across quartiles by Sex [file 12471_2025_2014_MOESM2_ESM.docx]

Fig S2

AFEQT overall summary score at baseline across quartiles by Sex
