# Supplementary material for: Quality of life before and after catheter ablation (pulmonary vein isolation) for atrial fibrillation: Results from the Netherlands Heart Registration
Source: Neth Heart J. 2026 Jan 19;34(2):72–9. doi: 10.1007/s12471-025-02014-6 (PMC12852550; doi:10.1007/s12471-025-02014-6)
Supplement: Supplementary file 3 — Tab S1. Baseline characteristics for patients with/without AFEQT data [file 12471_2025_2014_MOESM3_ESM.docx]

Table S1: Baseline characteristics for Patients with/without AFEQT data

| **Characteristics** | **Patients with AFEQT data**  **(evaluable cohort)**  **N= 2,534** | **Patients without AFEQT data**  **N= 8,002** |
| --- | --- | --- |
| **Patient Characteristics** | |  |
| Age, years Median (Q1,Q3) | 64 (57,70) | 63 (56,70) |
| < 65 years | N=1,342 (53%) | N=4,348 (54.8%) |
| ≥ 65 to 74 years | N=1,006 (39.7%) | N=2,998 (37.5%) |
| ≥ 75 years | N=186 (7.3%) | N=620 (7.7%) |
| Male Sex | N=1,666 (65.7%) | N=5,400 (67.5%) |
| BMI (kg/M2) Median (Q1, Q3) | 27 (24, 29) | 27 (25, 30) |
| **Medical history** | |  |
| Left ventricular ejection fraction (LVEF) ≥ 50% | N=2,039/2,341 (87.1%) | N=5,870/6,807 (86.2%) |
| Pre-operative moderate/severe mitral valve regurgitation | N=89/2,247 (4%) | N=414/6,682 (6.2%) |
| CHA₂DS₂-VASc Median (Q1,Q3) | 1 (1,2) | 1 (1,3) |
| 0-1 | N=1,254/2,497 (50.3%) | N=3,782/7,528 (50.2%) |
| 2 | N=662/2,497 (26.5%) | N=1,829/7,528 (24.3%) |
| 3 | N=352/2,497 (13.9%) | N=1,165/7,528 (15.5%) |
| 4 | N=156/2,497 (6.2%) | N=495/7,528 (6.6%) |
| ≥5 | N=73/2,497 (2.9%) | N=257/7,528 (3.4%) |
| Prior catheter ablation for AF | N=558/2,497 (22.3%) | N=1,799/7,569 (23,8%) |
| **Arrhythmia history** | |  |
| Type of AF at baseline |  |  |
| Paroxysmal | N=1,918/2,489 (77.1%) | N=5,304/7,508 (70,6%) |
| Persistent/longstanding persistent | N=571/2,489 (22.9%) | N=2,204/7,508 (29.4%) |
| **Treatment strategy** | |  |
| Point-by-point | N=1,076/2,487 (43.3%) | N=2,769/7,541 (36.7%) |
| PVAC ± MASC/MAAC* | N=499/2,487 (20.1%) | N=793/7,541 (10.5%) |
| Cryo-ballon | N=912/2,487 (36.7%) | N=3,979/7,541 (52.8%) |
| Additional LA ablation | N=306/2,340 (13.1%) | N=610/6,212 (9.8%) |

**multi-array septal catheter (MASC), multi-array ablation catheter (MAAC)*
