# Supplementary material for: Quality of life before and after catheter ablation (pulmonary vein isolation) for atrial fibrillation: Results from the Netherlands Heart Registration
Source: Neth Heart J. 2026 Jan 19;34(2):72–9. doi: 10.1007/s12471-025-02014-6 (PMC12852550; doi:10.1007/s12471-025-02014-6)
Supplement: Supplementary file 4 — Tab S2 Differences between patients in the lowest- and highest quartile AFEQT at baseline [file 12471_2025_2014_MOESM4_ESM.docx]

Tab S2 Differences between patients in the lowest- and highest quartile AFEQT at baseline

| **Characteristics** | **Patients in lowest quartile BL ≤41,18**  **N= 628** | **Patients in two middle quartiles BL (41.67-69.79) N= 1264** | **Patients in highest quartile BL (≥70,37)**  **N= 642** |
| --- | --- | --- | --- |
| **Baseline Characteristics** | | | |
| Age, years Median (Q1,Q3) | 64 (58,70) | 64 (58,70) | 63 (57,69) |
| < 65 years (%) | 318/628 (50.6%) | 661/1264 (52.3%) | 363/642 (56.5%) |
| ≥ 65 to 74 years (%) | 259/628 (41.2%) | 512/1264 (40.5%) | 235/642 (36.6%) |
| ≥ 75 years (%) | 51/628 (8.1%) | 91/1264 (7.2%) | 44/642 (6.9%) |
| Female Sex (%) | 295/628 (47%) | 441/1264 (34.9%) | 132/642 (20.6%) |
| BMI (kg/M2) Median (Q1, Q3) | 27.7 (24.9, 30.9) | 26.7 (24.4, 29.4) | 25.8 (24.2, 28.3) |
| **Medical history** | | | |
| LVEF ≥ 50 (%) | 484/582 (83.2%) | 1026/1264 (88%) | 529/593 (89.2%) |
| Pre-operative moderate/severe mitral valve regurgitation (%) | 19/559 (3.4%) | 54/1131 (4.8%) | 16/557 (2.9%) |
| CHA₂DS₂-VASc Median (Q1,Q3) | 2 (1,3) | 2 (1,2) | 1 (0,2) |
| 0-1 (%) | 257/615 (41.8%) | 610/1248 (48.9%) | 387/634 (61%) |
| 2 (%) | 160/615 (26%) | 344/1248 (27.6%) | 158/634 (24.9%) |
| 3 (%) | 115/615 (18.7%) | 183/1248 (14.7%) | 54/634 (8.5%) |
| 4 (%) | 56/615 (9.1%) | 75/1248 (6%) | 25/634 (3.9%) |
| ≥5 (%) | 27/615 (4.4%) | 36/1248 (2.9%) | 10/634 (1.6%) |
| Prior catheter ablation for AF (%) | 146/615 (23.7%) | 265/1247 (21.3%) | 147/635 (23.1%) |
| **Type of AF at baseline** | | | |
| Paroxysmal (%) | 456/613 (74.4%) | 943/1243 (75.9%) | 519/633 (82%) |
| Persistent/longstanding persistent (%) | 157/613 (25.6%) | 300/1243 (24.1%) | 114/633 (18%) |
| **Treatment strategy** | | | |
| Point-by-point (%) | 277/612 (45.3%) | 534/1243 (43%) | 265/632 (41.9%) |
| PVAC ± MASC/MAAC (%) | 136/612 (22.2%) | 253/1243 (20.4%) | 110/632 (17.4%) |
| Cryo-ballon (%) | 199/612 (32.5%) | 456/1243 (36.7%) | 257/632 (40.7%) |
| Additional LA ablation (%) | 68/572 (11.9%) | 161/1177 (13.7%) | 77/591 (13%) |
