# Supplementary material for: Quality of life before and after catheter ablation (pulmonary vein isolation) for atrial fibrillation: Results from the Netherlands Heart Registration
Source: Neth Heart J. 2026 Jan 19;34(2):72–9. doi: 10.1007/s12471-025-02014-6 (PMC12852550; doi:10.1007/s12471-025-02014-6)
Supplement: Supplementary file 5 — Tab S3: AFEQT en Delta AFEQT scores to quartile AFEQT at baseline [file 12471_2025_2014_MOESM5_ESM.docx]

Tab S3: AFEQT en Delta AFEQT scores to quartile AFEQT at baseline

| **Characteristics** | **Patients in lowest quartile BL ≤41.18**  **N= 628** | **Patients in two middle quartiles BL (41.67-69.79) N= 1264** | **Patients in highest quartile BL (≥70.37)**  **N= 642** |
| --- | --- | --- | --- |
| **Absolute scores** | | | |
| AFEQT at baseline (SD) | 30.4 (8.3) | 55.2 (8.1) | 81.2 (8.1) |
| AFEQT at one-year (SD) | 68.7 (23.7) | 80.3 (18.6) | 89.8 (12.4) |
| Delta AFEQT (SD) | 38.3 (23.6) | 25.1 (18.8) | 8.6 (13.3) |
| **Absolute change** | | | |
| Increased Delta AFEQT (%) | N= 585 (93.2%) | N= 1122 (88.8%) | N= 504 (78.5%) |
| Unchanged Delta AFEQT (%) | N= 4 (0.6%) | N=10 (0.8%) | N=12 (1.9%) |
| Decreased Delta AFEQT (%) | N= 39 (6.2%) | N=132 (10.4%) | N=126 (19.6%) |
