# Supplementary material for: Quality of life before and after catheter ablation (pulmonary vein isolation) for atrial fibrillation: Results from the Netherlands Heart Registration
Source: Neth Heart J. 2026 Jan 19;34(2):72–9. doi: 10.1007/s12471-025-02014-6 (PMC12852550; doi:10.1007/s12471-025-02014-6)
Supplement: Supplementary file 6 — Tab S4: Factors independently associated with dichotomized HRQoL outcomes at one-year follow-up [file 12471_2025_2014_MOESM6_ESM.docx]

Tab S4: Factors independently associated with dichotomized HRQoL outcomes at one-year follow-up

|  | **Results of Multivariable logregression model** | | | | |
| --- | --- | --- | --- | --- | --- |
|  | **No clinical important difference (Delta AFEQT < 5)** | |  | **Impaired HRQoL (AFEQT T1 <80)** | |
| **Factor** | **Odds ratio (95% CI)** | **P value** |  | **Odds ratio (95% CI)** | **P value** |
| Age  (per 1-year increase) | NS | NS |  | 1.016  (1.002 - 1.029) | .021 |
| Female  (vs male) | NS | NS |  | 1.423  (1.160 – 1,745) | <.001 |
| CHA₂DS₂-VASc 2  (vs 0-1) | 1.582  (1.215 – 2.060) | <.001 |  | 1.361  (1.065 – 1.741) | .014 |
| CHA₂DS₂-VASc 3  (vs 0-1) | 1.678  (1.196 – 2.355) | .003 |  | NS | NS |
| CHA₂DS₂-VASc 4  (vs 0-1) | 2.410  (1.545 – 3.760) | <.001 |  | NS | NS |
| CHA₂DS₂-VASc ≥5  (vs 0-1) | 3.482  (1.969 – 6.161) | <.001 |  | 2.103  (1.186 – 3.730) | .011 |
| Additional LA ablation (vs no) | 0.645  (0.431 – 0.965) | .033 |  | 0.709  (0.530 – 0.949) | .021 |
| Prior catheter ablation for AF (vs no) | 1.653  (1.256 – 2.176) | <.001 |  | 1.380  (1.092 – 1.743) | .007 |
| BMI (per 1-point increase) | NS | NS |  | 1.027  (1.004 – 1.050) | .020 |
| BL AFEQT (per 1-point increase) | 1.041  (1.035 – 1.048) | <.001 |  | 0.963 (0.958 - 0.968) | <.001 |
